# RETRACTED: Vaccaro et al. Differential Expression of Nitric Oxide Synthase Isoforms nNOS and iNOS in Patients with Non-Segmental Generalized Vitiligo. *Int. J. Mol. Sci.* 2017, *18*, 2533

**DOI:** 10.3390/ijms26178250

**Published:** 2025-08-26

**Authors:** Mario Vaccaro, Natasha Irrera, Giuseppina Cutroneo, Giuseppina Rizzo, Federico Vaccaro, Giuseppe P. Anastasi, Francesco Borgia, Serafinella P. Cannavò, Domenica Altavilla, Francesco Squadrito

**Affiliations:** 1 Department of Clinical and Experimental Medicine, University of Messina, Via Consolare Valeria, 98124 Messina, Italy; gcutroneo@unime.it (G.C.); fborgia@unime.it (F.B.); cannavop@unime.it (S.P.C.); fsquadrito@unime.it (F.S.); 2 Department of Biomedical Sciences and Morpho-Functional Images, University of Messina, I-98125 Messina, Italy; rizzog@unime.it (G.R.); feddy.fv@outlook.com (F.V.); anapuc@unime.it (G.P.A.); daltavilla@unime.it (D.A.)

The *International Journal of Molecular Sciences* retracts the article, “Differential Expression of Nitric Oxide Synthase Isoforms nNOS and iNOS in Patients with Non-Segmental Generalized Vitiligo” [1], cited above.

Following publication, concerns were brought to the attention of the publisher regarding an overlap in images between this article [1] and two other publications [2,3] produced by a similar authorship group.

Adhering to our standard procedure, the Editorial Office and Editorial Board conducted an investigation that confirmed an image duplication between Figure 5 in [1] and Figure 2 in [2,3], presenting different experimental conditions. While the authors cooperated with the Editorial Office during the investigation, they were unable to satisfactorily explain the overlap nor provide raw material of an acceptable standard for Editorial Board evaluation. As a result, the Editorial Board has lost confidence in the reliability of the findings and has decided to retract this publication [1], as per MDPI’s retraction policy (https://www.mdpi.com/ethics#_bookmark30, accessed on 14 July 2025).

This retraction was approved by the Editor-in-Chief of the *International Journal of Molecular Sciences*.

The authors did not provide a comment on this decision.
